# Comparison of the clinical efficacies of two L‐asparaginase‐based chemotherapy regimens for newly diagnosed nasal‐type extranodal NK/T‐cell lymphoma

**DOI:** 10.1002/cam4.5708

**Published:** 2023-03-31

**Authors:** Wanchun Wu, Kexin Ren, Xi Chen, Na Li, Qian Luo, Tao Hai, Huijie Zhou, Liqun Zou

**Affiliations:** ^1^ Department of Medical Oncology of Cancer Center West China Hospital, Sichuan University Chengdu China

**Keywords:** clinical efficacy, extranodal NK‐T‐cell lymphoma, L‐asparaginase, prognoses, survival, toxicity

## Abstract

**Background:**

Nasal‐type extranodal natural killer (NK)/T cell lymphoma (ENKTL) is a rare and aggressive type of lymphoma. The optimal chemotherapy regimen for ENKTL has not yet been established. In this study, we compared the LVDP (L‐asparaginase, etoposide, dexamethasone, and cisplatin) and GLIDE (gemcitabine, L‐asparaginase, ifosfamide, dexamethasone, and etoposide) chemotherapy regimens for the treatment of ENKTL.

**Methods:**

A total of 267 patients with newly diagnosed ENKTL were included in this retrospective study. Propensity score matching (PSM) was used to adjust for confounders between the LVDP and GLIDE groups. Treatment responses, survival outcomes, and toxicities between the two groups were compared before and after PSM.

**Results:**

At the end of therapy, the objective response rate (ORR) and complete response (CR) were 83.5% and 62.2%, respectively, for all patients. The ORR and CR were 85.5% and 62.2% for the LVDP group compared with 79.3% and 62.2% for the GLIDE group, respectively, and no differences between the two groups were found (ORR, *p* = 0.212; CR, *p* = 0.996). With a median 71 months follow‐up, the 5‐year progression‐free survival (PFS) and overall survival (OS) rates were 64.3% and 68.5%, respectively. The 5‐year PFS and OS were 65.6% and 70.1% for the LVDP group compared with 61.6% and 64.6% for the GLIDE group, respectively (PFS, *p* = 0.478; OS, *p* = 0162). After PSM, no significant differences in short‐term efficacy (ORR, *p* = 0.696; CR, *p* = 0.264) or long‐term efficacy (PFS, *p* = 0.794; OS, *p* = 0.867) between the two groups were identified. However, treatment‐related toxicities were milder in the LVDP group compared to the GLIDE group, even after adjusting for confounders via PSM.

**Conclusion:**

In conclusion, both LVDP and GLIDE regimens are effective for the treatment of ENKTL. However, the LVDP regimen is safer than the GLIDE regimen, with milder treatment‐related toxicities. Therefore, the LVDP regimen could be a preferable option for patients with ENKTL.

## Graphical abstract

In this retrospective analysis of 267 newly diagnosed nasal‐type extranodal NK/T‐cell lymphoma patients, LVDP (L‐asparaginase, etoposide, dexamethasone and cisplatin) and GLIDE (gemcitabine, L‐asparaginase, ifosfamide, dexamethasone and etoposide) chemotherapy regimens showed similar short‐term and long‐term efficacies, with 85.5% ORR and 62.2% CR rates, and 64.3% PFS and 68.5% OS rates at a median 71‐month follow‐up. However, LVDP was associated with milder treatment‐related toxicities compared to GLIDE, even after adjusting for confounders via PSM. These findings suggest that LVDP could be a preferable option for patients with nasal‐type extranodal NK/T‐cell lymphoma.

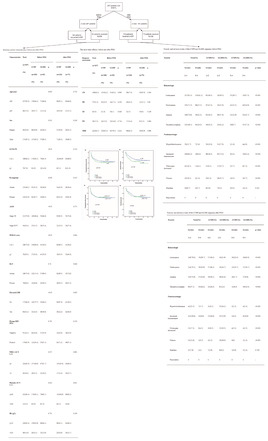

## 1 INTRODUCTION

Extranodal Natural killer (NK)/T cell lymphoma (ENKTL) is an aggressive non‐Hodgkin lymphoma (NHL) associated with Epstein–Barr virus (EBV), with a distinctive geographic distribution and a high incidence in East Asia and Latin America.^1^, ^2^, ^3^ In China, approximately 12% of malignant lymphomas, and nearly half of mature T‐cell and NK‐cell lymphomas are ENKTL.^4^ Moreover, 80% of ENKTL patients with the upper aerodigestive tract involvement at initial diagnosis, including nasal cavity, nasopharynx, paranasal sinuses, and palate involvement, which are defined as nasal‐type ENKTL.

In the past, anthracycline‐based chemotherapy was conventionally administered for patients with ENKTL, including CHOP (cyclophosphamide, doxorubicin, vincristine, and prednisone) and CHOP‐like, while the survival outcomes of those regimens were poor, achieving 5‐year progression‐free survival (PFS) and overall survival (OS) rates of 41.6%–47.6% and 54.5%–60.6%, respectively.^5^ As a result, L‐asparaginase‐based regimens were developed and produced improved survival rates. Among these newly established regimens were LVDP (L‐asparaginase, etoposide, dexamethasone, and cisplatin), GLIED (gemcitabine, L‐asparaginase, ifosfamide, dexamethasone, and etoposide), SMILE (dexamethasone, methotrexate, ifosfamide, L‐asparaginase, and etoposide), P‐Gemox (pegaspargase, gemcitabine, and oxaliplatin), and DDGP (dexamethasone, cisplatin, gemcitabine, and pegaspargase).^6^, ^7^, ^8^, ^9^, ^10^ However, currently, there is no optimal chemotherapy regimen for ENKTL patients because of the low incidence of this disease.

One clinical study reported on the administration of GLIDE for newly diagnosed ENKTL patients, and with a 2‐year median follow‐up, the estimated 3‐year OS was 56%.^6^ However, severe myelosuppression led to severe chemotherapy‐related infections. Approximately 75.9% of patients underwent grade 3/4 neutropenia. In another study, 66 patients with ENKTL received the LVDP regimen with a median follow‐up of 23.5 months, the 3‐year PFS rate was 67.4%, and the OS rate was 70.1%.^7^ However, there are no data comparing the clinical efficacy, chemotherapy‐related toxicity, and survival outcomes of LVDP versus GLIDE regimens in newly diagnosed ENKTL. Therefore, we conducted this retrospective study to explore the long‐term efficacy and toxicities of LVDP and GLIDE chemotherapy regimens for patients with ENKTL.

## 2 METHOD

### 2.1 Patients

A retrospective analysis for patients with newly diagnosed ENKTL was carried out in our investigation. The following criteria were used for patient inclusion: (1) confirmed as ENKTL from January 2012 to January 2017 in accordance with the 2008 WHO lymphoma classification;^11^ (2) had 2 cycles or more of LVDP or GLIDE treatment; (3) adequate clinical data and follow‐up information for survival analysis; (4) no other malignant tumors; (5) no previous antitumor treatment. Patients with nonnasal‐type ENKTL were excluded. Ethical approval for this study was granted by the ethics committee of West China Hospital (ID of ethics approval: SCHX‐2022‐64).

Patients' baseline characteristics and pre‐treatment laboratory data were obtained for analysis, including age, gender, Eastern Cooperative Oncology Group performance status (ECOG) score, B symptoms, white blood count (WBC), Hb (Hemoglobin), platelet count, lactate dehydrogenase (LDH), plasma EBV DNA, and bone marrow examination. Imaging examination was used for staging, including computed tomography (CT) and positron emission tomography/computed tomography (PET/CT). Response assessment was also carried out using PET/CT. However, some patients chose CT for response assessment due to individual economic circumstances. The PINK‐E scoring system was utilized in this study. Regional lymph node (RLN) involvement was classified as N1, N2, or N3, according to the definitions of the tumor‐node‐metastasis (TNM) staging system.

### 2.2 Treatment

In this study, 267 patients with ENKTL were included. Among these, 63 patients were treated through chemotherapy only, and 204 patients were treated by chemoradiotherapy. Within this group, LVDP combined with radiotherapy was administered to 166 patients, and GLIDE combined with radiotherapy was given to 38 patients. The LVDP regimen was repeatedly administered every 21 days in this way: L‐asparaginase (5500 IU/m^2^ intravenously on days 1–5), etoposide (80 mg/m^2^ intravenously on days 1–3), dexamethasone (40 mg/day intravenously on days 1–4), and cisplatin (25 mg/m^2^ intravenously on days 1–3). An L‐asparaginase skin test administered to each patient prior to each cycle; if the result was positive, pegaspargase replaced L‐asparaginase on day 4. The schedule and dose of the GLIDE regimen were given as described in a previous study.^6^

Patients with confirmed early‐stage ENKTL were treated by chemotherapy combined with involved‐field radiation therapy (IFRT), and in our study, 191 of 213 early‐stage patients received radiotherapy. Patients with confirmed advanced‐stage ENKTL were treated by consolidation radiation therapy at the primary tumor site or local residual lesion following the completion of the patient's chemotherapy schedule. However, only 13 of 54 advanced patients received radiation therapy consolidation after chemotherapy. Moreover, patients with complete response (CR) or partial response (PR) after initial treatment were allowed to receive elective hematopoietic stem cell transplantation (HSCT). Owing to a lack of standardized treatment protocols, treatment regimens varied and were decided primarily by physician discretion.

### 2.3 Response assessment

Treatment responses of all patients were assessed according to the response criteria of the Lugano Classification, which included the following terms: CR, PR, stable disease (SD), and progressive disease (PD).^12^ The objective response rate (ORR) referred to the proportion of patients who have a CR or PR. OS was calculated from the initial diagnosis to death or the last follow‐up. PFS was used to refer to the time from the initial diagnosis to disease recurrence, progression, or any‐cause death. OS following the first disease relapse or progression was calculated from relapse or progression to death or the last follow‐up.

### 2.4 Toxicity assessment

The 5th version of the Common Terminology Criteria for Adverse Events (CTCAE) was used for toxicity evaluation. All toxicity data were obtained via physical examinations and blood tests including complete blood cell count, kidney, and liver tests.

### 2.5 Statistical analysis

All categorical variables are displayed in the form of frequencies with associated percentages, and associations between categorical variables were elucidated through the chi‐square test. The Kaplan–Meier method was employed to calculate survival results, and the log‐rank test was conducted to compare survival curves. Cox regression analyses were carried out to determine independent prognostic variables for ENKTL. Two‐sided P‐values less than 0.05 were considered statistically significant.

We performed nearest‐neighbor 1:2 propensity score matching (PSM) (caliper = 0.02) without replacement to adjust confounding factors between the LVDP and GLIDE groups. Covariates were considered well‐balanced when the absolute value of the standardized difference was lower than 10%. After PSM, baseline covariates and survival outcomes were compared between chemotherapy groups. PSM was conducted by SPSS 22 (IBM Corp). SPSS was used for performing all statistical analyses and for constructing visual figures.

## 3 RESULTS

### 3.1 Patients' baseline characteristics

A total of 267 patients with ENKTL were included in this study. Within this cohort, 185 patients received LVDP and 82 patients received GLIDE. Fourty‐three years was the median age of patients (range, 13–76 years), with 227 (85%) patients less than 60 years, and 174 (65.2%) patients were male (male/female ratio, 1.9:1). Most patients (92.1%) had ECOG PS scores of 0 or 1. Approximately 144 (53.9%) patients had B symptoms. Seventy‐eight (29.2%) patients presented with RLN. Most patients (70.8%) were classified as low‐risk (0 or 1) according to the PINK‐E system. Elevated serum LDH levels were detected for 94 (35.2%) patients, and 176 (65.9%) patients were positive for EBV‐DNA. Before PSM, there were significant differences in B symptoms, AASS, PINK‐E, ECOG PS, and LDH between the LVDPand GLIDEgroups. However, after 1:2 PSM at a caliper value of 0.02, no significant differences in clinical parameters between the LVDP and GLIDE groups were identified (LVDP 118 cases, GLIDE 73 cases). Baseline characteristics for included patients are presented in Table 1.

**TABLE 1:** Baseline patient characteristics before and after PSM.

Characteristics	Total (*n* = 267) (%)	Before PSM			After PSM		
		LVDP (*n* = 185) (%)	GLIDE (*n* = 82) (%)	*p*	LVDP (*n* = 118) (%)	GLIDE (*n* = 73) (%)	*p*
Age (year)				0.633			0.732
<60	227 (85.0)	156 (84.3)	71 (86.6)		98 (83.1)	62 (84.9)	
≥60	40 (15.0)	29 (15.7)	11 (13.4)		20 (16.9)	11 (15.1)	
Sex				0.321			0.246
Female	93 (34.8)	68 (36.8)	25 (30.5)		47 (39.8)	23 (31.5)	
Male	174 (65.2)	117 (63.2)	57 (69.5)		71 (60.2)	50 (68.5)	
ECOG PS				<0.05			0.130
0 or 1	189 (92.1)	176 (95.1)	70 (85.4)		112 (94.9)	65 (89.0)	
≥2	78 (7.9)	9 (4.9)	12 (14.6)		6 (5.1)	8 (11.0)	
B symptoms				0.039			0.315
Absent	123 (46.1)	93 (50.3)	30 (36.6)		54 (45.8)	28 (38.4)	
Present	144 (53.9)	92 (49.7)	52 (63.4)		64 (54.2)	45 (61.6)	
AASS				<0.05			0.571
Stage I/II	213 (79.8)	160 (86.4)	53 (64.6)		95 (80.5)	53 (72.6)	
Stage III/IV	54 (20.2)	25 (13.5)	29 (35.4)		23 (19.5)	20 (27.4)	
PINK‐E score				<0.05			0.062
0 or 1	189 (70.8)	148 (80.0)	41 (50.0)		82 (69.5)	41 (56.2)	
≥2	78 (29.2)	37 (20.0)	41 (50.0)		36 (30.5)	32 (43.8)	
RLN				0.76			0.646
Absent	189 (70.8)	132 (71.4)	57 (69.5)		82 (69.5)	53 (72.6)	
Present	78 (29.2)	53 (28.6)	25 (30.5)		36 (30.5)	20 (27.4)	
Elevated LDH				<0.05			0.065
No	173 (64.8)	140 (75.7)	33 (40.2)		80 (67.8)	41 (56.2)	
Yes	94 (35.2)	45 (24.3)	49 (59.8)		38 (32.2)	32 (43.8)	
Plasma EBV‐DNA				0.791			0.553
Negative	91 (34.1)	64 (34.6)	27 (32.9)		34 (28.8)	24 (32.9)	
Positive	176 (65.9)	121 (65.4)	55 (67.1)		84 (71.2)	49 (67.1)	
WBC (×10^9^ L^−1^)				0.517			0.891
≥4	224 (83.9)	157 (84.9)	67 (81.7)		101 (85.6)	63 (86.3)	
<4	43 (16.1)	28 (15.1)	15 (18.3)		17 (14.4)	10 (13.7)	
Platelet (×10^9^ L^−1^)				0.422			0.611
≥100	252 (94.4)	176 (95.1)	76 (92.7)		112 (94.9)	68 (93.2)	
<100	15 (5.4)	9 (4.9)	6 (7.3)		6 (5.1)	5 (6.8)	
Hb (g/L)				0.701			0.549
≥110	228 (85.4)	159 (85.9)	69 (84.1)		98 (83.1)	63 (86.3)	
<110	39 (14.6)	26 (14.1)	13 (15.9)		20 (16.9)	10 (13.7)	

Abbreviations: AASS, Ann Arbor staging system; EBV, Epstein–Barr virus; ECOG PS, Eastern Cooperative Oncology Group performance status; GLIDE, gemcitabine, L‐asparaginase, ifosfamide, dexamethasone, and etoposide; Hb, hemoglobin; LDH, lactate dehydrogenase; LVDP, L‐asparaginase, etoposide, dexamethasone and cisplatin; PINK‐E, prognostic index of natural killer lymphoma with EBV; RLN, regional lymph node; WBC, white blood cell.

### 3.2 The short‐term efficacy of the LVDP and GLIDE regimens

Patients received a median of four (range, 2–8) cycles of chemotherapy. The entire cohort was assessed (230 of 267 patients were assessed by PET‐CT), including 166 (62.2%) CRs, 57 (21.3%) PRs, 8 (3.0%) SDs, and 36 (13.5%) PDs, with an ORR of 83.5%. Twenty patients received upfront autoHSCT after achieving CR. Before PSM, the CR and ORR for patients who received the LVDP regimen were 62.2% and 85.5%, respectively, and the CR and ORR for patients who received the GLIDE regimen were 62.2% and 79.3%, respectively. No significant differences between the LVDP and GLIDE groups were identified (CR, *p* = 0.996; ORR, *p* = 0.212). After PSM, there were 68 (57.6%) CRs, 30 (25.4%) PRs, 3 (2.5%) SDs, and 17 (14.4%) PDs for the LVDP regimen, and 48 (65.8%) CRs, 11 (15.1%) PRs, 5 (6.8%) SDs, and 9 (12.3%) PDs for the GLIDE regimen. The ORRs for the LVDP and GLIDE regimens were 83.0% and 80.9%, respectively. Similarly, no difference in short‐term efficacy between the two groups was found (Table 2). In addition, by comparing the efficacy of the two regimens for early‐stage and advanced‐stage patients, it was found that the GLIDE regimen had a higher CR rate for advanced‐stage patients (Table S1).

**TABLE 2:** The short‐term efficacy before and after PSM.

Response assessment	Total (*n* = 267) (%)	Before PSM				After PSM	
		LVDP (*n* = 185) (%)	GLIDE (*n* = 82) (%)	*p*	LVDP (*n* = 118) (%)	GLIDE (*n* = 73) (%)	*p*
CR	166 (62.2)	115 (62.2)	51 (62.2)	0.996	68 (57.6)	48 (65.8)	0.264
PR	57 (21.3)	43 (23.3)	14 (17.1)	0.256	30 (25.4)	11 (15.1)	0.09
SD	8 (3.0)	3 (1.6)	5 (6.1)	0.048	3 (2.5)	5 (6.8)	0.149
PD	36 (13.5)	24 (13.0)	12 (14.6)	0.714	17 (14.4)	9 (12.3)	0.684
ORR	223 (83.5)	158 (85.5)	65 (79.3)	0.212	98 (83.0)	59 (80.9)	0.696

Abbreviations: CR, complete response; GLIDE, gemcitabine, L‐asparaginase, ifosfamide, dexamethasone, and etoposide; LVDP, L‐asparaginase, etoposide, dexamethasone and cisplatin; ORR, objective response rate; PD, progressive disease; PR, partial response; SD, stable disease.

### 3.3 The long‐term efficacy of the LVDP and GLIDE regimens

**FIGURE 1:**
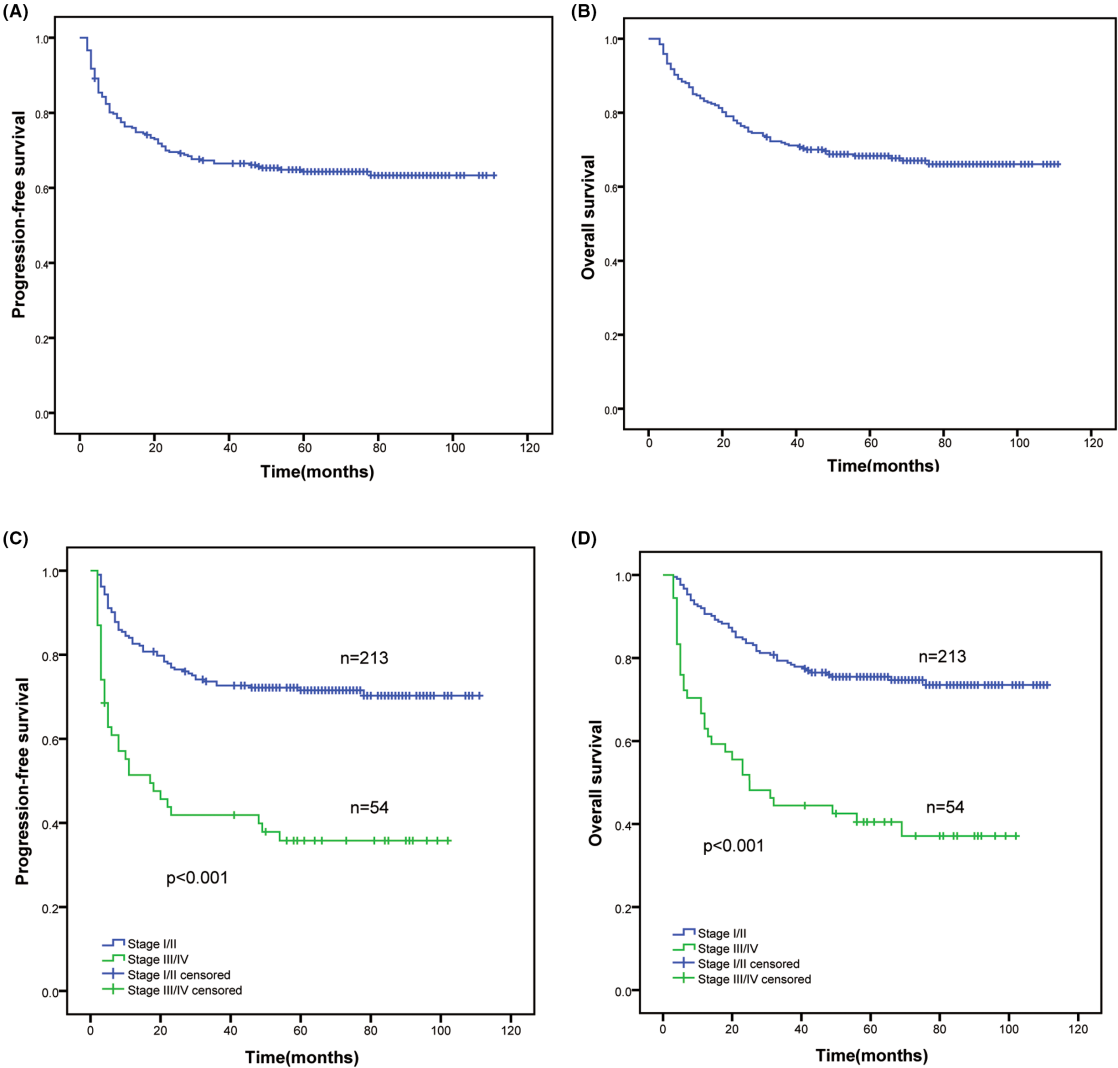
The long‐term survival outcomes for all patients with ENKTL. (A, B) PFS and OS in all patients; (C, D) PFS and OS in early‐stage and advanced ENKTL. ENKTL, extranodal NK‐T‐cell lymphoma; OS, overall survival; PFS, progression‐free survival.

Follow‐ups were conducted for all 267 patients after a median of 71 months (95% confidence interval [CI], 68.2–73.8), and the median survival time (including PFS and OS) was not reached. After the final follow‐up, 87 of 267 patients died. For 267 patients, the 3‐year PFS and OS rates were 67.3% and 72.1%, respectively, and the 5‐year PFS and OS rates were 64.3% and 68.5%, respectively (Figure 1A,B). The 3‐ and 5‐year PFS rates of early‐stage patients were 73.1% and 71.5%, respectively. In comparison with early‐stage patients, the PFS rates of advanced‐stage patients were significantly lower (3‐year PFS rate, 41.9%; 5‐year PFS, 35.8%) (*χ* ^2^ = 32.919, *p* < 0.001) (Figure 1C). Likewise, for early‐stage patients, the OS rates (3‐year OS, 78.9%; 5‐year OS, 75.5%) were substantially higher than the OS rates of advanced‐stage patients (3‐year OS, 44.4%; 5‐year OS, 40.5%) (*χ* ^2^ = 35.280, *p* < 0.001) (Figure 1D).

**FIGURE 2:**
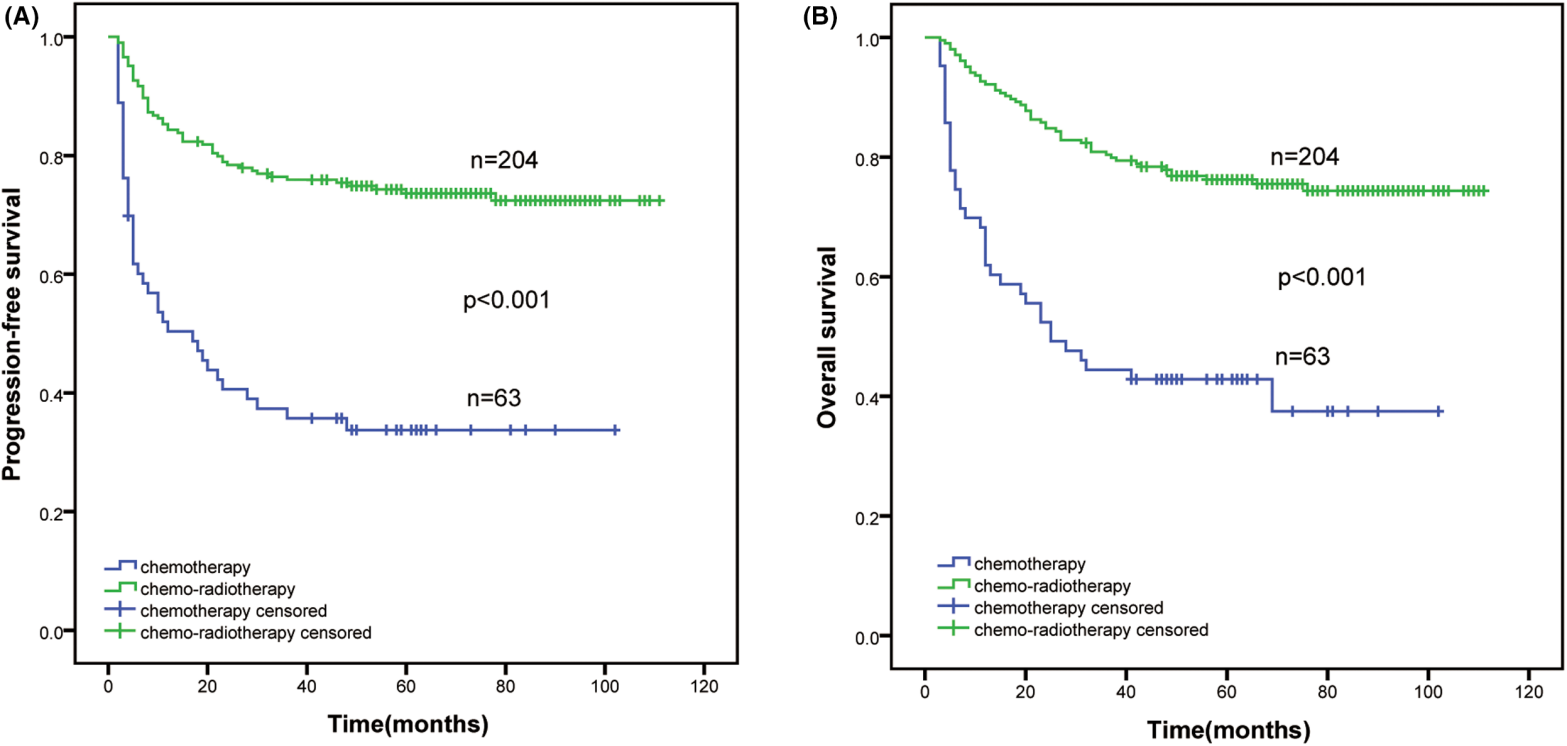
The long‐term survival of different treatment methods for ENKTL. (A, B) PFS and OS in the chemotherapy and chemoradiotherapy groups. ENKTL, extranodal NK‐T‐cell lymphoma; OS, overall survival; PFS, progression‐free survival.

Approximately 76.4% of patients received chemoradiotherapy, and their PFS and OS rates were higher than those of patients who were administered chemotherapy alone (PFS, *χ* ^2^ = 46.514, *p* < 0.001; OS, *χ* ^2^ = 38.480, *p* < 0.001; Figure 2A,B). For the chemo‐radiotherapy group, the median survival time was not reached; the rates of 3‐year PFS and OS were 75.9% and 80.4%, respectively; and the rates of 5‐year PFS and OS were 73.6% and 76.2%, respectively. However, for the chemotherapy‐alone group, the median survival time had been reached at 17 months (95% CI, 5.5–28.3 months) for PFS and 25 months (95% CI, 11.7–38.3 months) for OS; the rates of 3‐ year PFS and OS were 35.7% and 43.7%, and the rates of 5‐year PFS and OS were 33.7% and 42.9%, respectively (Figure 2A,B).

**FIGURE 3:**
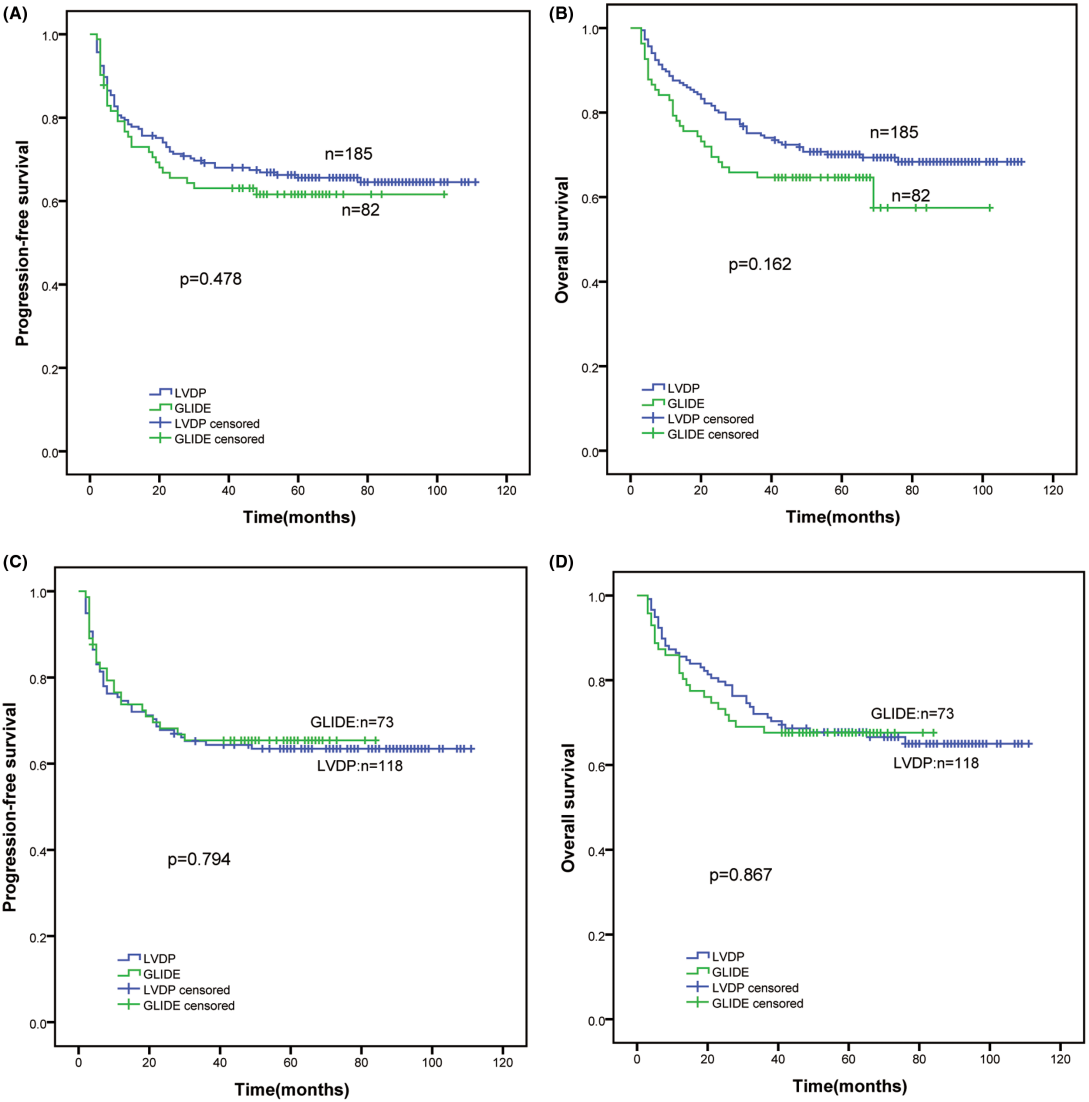
Before and after PSM, the long‐term survival of LVDP and GLIDE regimens for ENKTL. (A, B) Before PSM, PFS, and OS in the LVDP and GLIDE groups; (C, D) after PSM, PFS, and OS in the LVDP and GLIDE groups. ENKTL, extranodal NK‐T‐cell lymphoma; GLIDE, gemcitabine, L‐asparaginase, ifosfamide, dexamethasone, and etoposide; LVDP, L‐asparaginase, etoposide, dexamethasone, and cisplatin; OS, overall survival; PFS, progression‐free survival; PSM, propensity score matching.

Before PSM, for the LVDP group, the median survival time was not reached. The rates of 3‐year PFS and OS were 69.1% and 74.3%, respectively, and the rates of 5‐year PFS and OS were 65.6% and 70.1%, respectively (Figure 3A,B). Likewise, for the GLIDE group, the median survival time was also not reached. The rates of 3‐year PFS and OS were 63.1% and 64.6%, respectively; and the rates of 5‐year PFS and OS were 61.6% and 64.6%, respectively (Figure 3A,B). No statistically significant differences between the LVDP and GLIDE groups were identified for PFS (*χ* ^2^ = 0.504, *p* = 0.478) or OS (*χ* ^2^ = 1.958, *p* = 0.162) (Figure 3A,B). After PSM, for the LVDP group, the median survival time was not reached. The rates of 3‐year PFS and OS were 64.4% and 71.4%, respectively; and 5‐year PFS and OS were 63.5% and 67.7%, respectively (Figure 3C,D). Similarly, for the GLIDE group, the median survival time was also not reached. The rates of 3‐year PFS and OS were 65.4% and 67.6%, respectively; these rates remained identical for 5‐year PFS and OS (Figure 3C,D). No statistically significant differences between the LVDP and GLIDE groups were discovered in PFS (*χ* ^2^ = 0.068, *p* = 0.794) or OS (*χ* ^2^ = 0.028, *p* = 0.867) (Figure 3C,D).

### 3.4 Survival results of patients with relapse or progression

**FIGURE 4:**
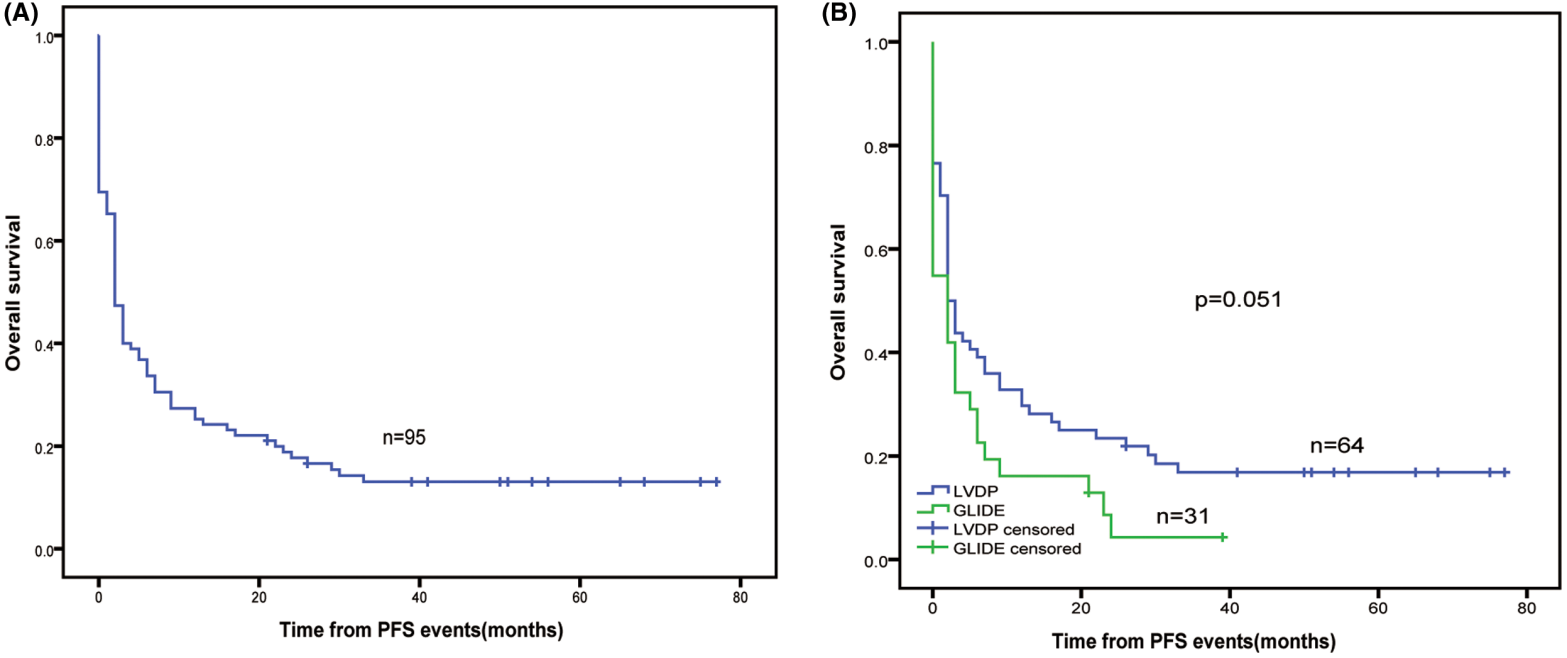
OS of patients with ENKTL after first relapse or progression. A, OS after first relapse or progression in all patients; B, OS after first relapse or progression in the LVDP and GLIDE groups. ENKTL, extranodal NK‐T‐cell lymphoma; GLIDE, gemcitabine, L‐asparaginase, ifosfamide, dexamethasone, and etoposide; LVDP, L‐asparaginase, etoposide, dexamethasone, and cisplatin; OS, overall survival.

In total, 95 PFS events were recorded after the last follow‐up. During the upfront treatment, 36 cases had progressive disease. After the upfront treatment, 57 cases had disease relapse. For patients who experienced the first relapse or progression, the median OS was short with 2 months only, and the 5‐year OS rate was 13.1% (Figure 4A). The OS after the first relapse or progression of the LVDP group was slightly higher than that of the GLIDE group, and there was a borderline significant difference between the two groups (*χ* ^2^ = 3.833, *p* = 0.051; Figure 4B). After the final follow‐up, 13 cases remained disease‐free after the first relapse or progression. Among these, four patients who received SMILE chemotherapy underwent auto‐HSCT, six patients underwent P‐Gemox combined with IFRT, and three patients were given anti‐PD‐1 antibody as maintenance therapy after receiving P‐Gemox (*n* = 2) and SMILE (*n* = 1).

### 3.5 Prognostic factors

To investigate clinical variables and their associations with survival outcomes for the entire cohort (267 patients), Cox regression analyses were conducted to determine independent prognostic factors. After the univariate Cox regression analyses, the clinical variables that may influence the PFS and OS of ENKTL patients are presented in Table 3. The parameters which were found to be related to the PFS of patients are listed as follows: ECOG PS, B symptoms, Elevated LDH, positive EBV‐DNA, RLN, Stage III/IV, PINK‐E ≥2, WBC (×10^9^ L^−1^) <4, and Hb level <110 g/L. With the exception of EBV‐DNA, all of the above parameters were found to have a correlation on patient OS.

**TABLE 3:** Univariate analysis of PFS and OS in 267 ENKTL patients.

	PFS			OS		
	HR	95% CI	*p*	HR	95% CI	*p*
Age >60	1.153	0.642–2.070	0.633	0.843	0.458–1.551	0.583
Male	0.724	0.466–1.126	0.152	1.418	0.891–2.256	0.141
ECOG PS ≥2	0.278	0.164–0.472	<0.001	0.278	0.158–0.482	<0.001
B symptoms	0.619	0.408–0.939	0.024	1.692	1.091–2.624	0.019
Elevated LDH	1.939	1.293–2.907	0.001	2.151	1.410–3.283	<0.001
EBV‐DNA positive	0.738	0.583–0.933	0.011	0.796	0.627–1.011	0.061
RLN	1.651	1.091–2.501	0.018	1.705	1.105–2.630	0.016
Stage III/IV	0.315	0.207–0.480	<0.001	0.292	0.189–0.450	<0.001
PINK‐E ≥2	0.351	0.234–0.527	<0.001	2.666	1.747–4.070	<0.001
WBC (×10^9^ L^−1^) < 4	0.545	0.338–0.878	0.013	0.570	0.346–0.940	0.028
Platelet (×10^9^ L^−1^) < 100	0.757	0.331–1.731	0.510	0.629	0.275–1.443	0.274
Hb <110 g/L	0.382	0.240–0.607	<0.001	0.480	0.291–0.791	0.004
LVDP	0.926	0.747–1.148	0.483	0.855	0.684–1.067	0.165

Abbreviations: EBV, Epstein–Barr virus; ECOG PS, Eastern Cooperative Oncology Group performance status; Hb, hemoglobin; LDH, lactate dehydrogenase; LVDP, L‐asparaginase, etoposide, dexamethasone and cisplatin; PINK‐E, prognostic index of natural killer lymphoma with EBV; RLN, regional lymph node; WBC, white blood cell.

To identify potential independent prognostic factors for PFS and OS, a multivariate analysis was conducted integrating all variables identified as significant in univariate analysis (Table 4). The results showed that ECOG PS, Stage III/IV, and Hb level <110 g/L were independent prognostic factors of PFS for ENKTL. In terms of OS, independent prognostic factors were ECOG PS and Stage III/IV.

**TABLE 4:** Multivariate analysis of PFS and OS in 267 ENKTL patients.

	PFS			OS		
	HR	95% CI	*p*	HR	95% CI	*p*
ECOG PS ≥2	1.897	1.008–3.052	0.047	2.040	1.061–3.923	0.032
B symptoms	1.000	0.626–1.599	1	1.131	0.692–1.849	0.623
Elevated LDH	0.970	0.586–1.606	0.906	1.180	0.701–1.986	0.533
EBV‐DNA positive	0.837	0.648–1.028	0.175	NA	NA	NA
RLN	1.565	0.999–2.452	0.059	1.433	0.902–2.278	0.128
Stage III/IV	1.800	0.994–2.452	0.041	2.142	1.164–3.942	0.014
PINK‐E ≥2	1.483	0.815–2.698	0.197	1.317	0.724–2.394	0.367
Hb <110 g/L	1.828	1.077–3.103	0.037	0.770	0.433–1.370	0.374
WBC (×10^9^ L^−1^) <4	0.915	0.532–1.574	0.748	0.969	0.542–1.734	0.916

Abbreviations: EBV, Epstein–Barr virus; ECOG PS, Eastern Cooperative Oncology Group performance status; Hb, hemoglobin; LDH, lactate dehydrogenase; PINK‐E, prognostic index of natural killer lymphoma with Epstein–Barr virus; RLN, regional lymph node; WBC, white blood cell.

### 3.6 Toxicity

Before PSM, treatment‐related toxicities, including hematologic and nonhematologic adverse events, are shown in Table 5. In this cohort, major adverse events (>25%) were noted for all patients, including myelosuppression and increased transaminase levels. Fifty‐eight patients presented with febrile neutropenia, and of these, 29 patients had infections, and 4 patients died of infection during the neutropenia period. No pancreatitis was reported. Compared with the GLIDE group, the rates of grade 3/4 hematologic toxicity (leukopenia, 29.7%; neutropenia, 22.2%; anemia, 12.9%; thrombocytopenia, 9.7%) were notably lower in the LVDP group. Additionally, the rates of grade 3/4 nonhematologic toxicity (hyperbilirubinemia, 1.6%; transaminase increased, 3.2%; fibrinogen decreased, 2.7%; nausea, 0.5%) were also lower in the LVDP group. After PSM, treatment‐related toxicities were listed in Table 6. The result was similar to the result before PSM. Except for diarrhea, the rates of both grade 3/4 hematologic and nonhematologic toxicity were lower in the LVDP group.

**TABLE 5:** Toxicity and adverse events of the LVDP and GLIDE regimens before PSM.

Toxicity	Total (%)		LVDP (%)	GLIDE (%)	LVDP (%)	GLIDE (%)	*p* value
	Grades 1–4	Grades 3–4	Grades 1–2	Grades 1–2	Grades 3–4	Grades 3–4	
Hematologic							
Leukopenia	217 (81.3)	110 (41.2)	91 (49.1)	16 (19.5)	55 (29.7)	55 (67.1)	<0.001
Neutropenia	191 (71.5)	86 (32.2)	87 (47.0)	18 (21.9)	41 (22.2)	45 (54.9)	<0.001
Anemia	189 (70.8)	59 (22.1)	91 (49.2)	39 (47.6)	24 (12.9)	35 (42.7)	<0.001
Thrombocytopenia	131 (49.1)	65 (24.3)	56 (30.3)	10 (12.2)	18 (9.7)	47 (57.3)	<0.001
Nonhematologic							
Hyperbilirubinemia	58 (21.7)	7 (2.6)	20 (10.8)	31 (37.8)	3 (1.6)	4 (4.9)	<0.001
Increased transaminase	166 (62.2)	16 (6.0)	89 (48.1)	61 (74.4)	6 (3.2)	10 (12.2)	<0.001
Fibrinogen decreased	43 (16.1)	11 (4.1)	15 (8.1)	17 (20.7)	5 (2.7)	6 (7.3)	<0.001
Nausea	43 (16.1)	4 (1.4)	10 (5.4)	26 (31.7)	1 (0.5)	3 (3.7)	<0.001
Diarrhea	18 (6.7)	2 (0.7)	9 (4.9)	7 (8.5)	1 (0.5)	1 (1.2)	0.425
Pancreatitis	0	0	0	0	0	0	—

**TABLE 6:** Toxicity and adverse events of the LVDP and GLIDE regimens after PSM.

Toxicity	Total (%)		LVDP (%)	GLIDE (%)	LVDP (%)	GLIDE (%)	*p* value
	Grades 1–4	Grades 3–4	Grades 1–2	Grades 1–2	Grades 3–4	Grades 3–4	
Hematologic							
Leukopenia	149 (78.0)	76 (39.7)	57 (48.3)	16 (21.9)	30 (25.4)	46 (63.0)	<0.001
Neutropenia	134 (70.2)	59 (30.9)	57 (48.3)	18 (24.7)	22 (18.7)	37 (50.7)	<0.001
Anemia	143 (74.9)	47 (24.6)	58 (49.1)	38 (42.0)	20 (1.7)	27 (9.6)	<0.001
Thrombocytopenia	90 (47.1)	50 (26.2)	31 (26.3)	9 (12.3)	11 (9.3)	39 (53.4)	<0.001
Nonhematologic							
Hyperbilirubinemia	43 (22.5)	7 (3.7)	11 (9.3)	25 (34.2)	3 (2.5)	4 (5.5)	<0.001
Increased transaminase	122 (58.6)	13 (6.8)	55 (46.6)	54 (73.9)	5 (4.2)	8 (10.9)	<0.001
Fibrinogen decreased	33 (17.3)	8 (4.2)	10 (8.5)	15 (20.5)	4 (3.3)	4 (5.5)	<0.001
Nausea	24 (12.6)	1 (0.5)	4 (3.3)	19 (26.0)	0 (0)	1 (1.4)	<0.001
Diarrhea	15 (7.9)	2 (1)	7 (5.9)	6 (8.2)	1 (0.8)	1 (1.4)	0.762
Pancreatitis	0	0	0	0	0	0	—

## 4 DISCUSSION

In this real‐world retrospective study, we analyzed two L‐asparaginase‐based regimens (LVDP and GLIDE) for 267 ENKTL patients, and we determined that the L‐asparaginase‐based chemotherapy regimens were effective. No differences in PFS or OS between the LVDP and GLIDE regimens were discovered, even after PSM. However, compared with the LVDP regimen, treatment‐related toxicities, including hematologic and nonhematologic adverse events, were more common in the GLIDE regimen. Through the Cox regression analysis, we found that ECOG PS ≥2 and Stage III/IV were correlated with poorer OS and PFS.

Recently, owing to the anthracycline resistance of ENKTL, the use of L‐asparaginase‐based regimens has been preferred for improved efficacy. The SMILE regimen was given in newly diagnosed ENKTL patients and achieved a 4‐year PFS of 64% and a 5‐year OS of 50%.^8^ The survival outcomes of GELOX (L‐asparaginase, gemcitabine, and oxaliplatin) and P‐Gemox from another retrospective study were evaluated for ENKTL patients and yielded a 3‐year OS of 65.2% and 3‐year PFS of 57%.^13^ In China, 165 patients from a prospective clinical trial were treated with P‐Gemox plus thalidomide or AspaMetDex (L‐asparaginase, methotrexate, and dexamethasone), and a 3‐year PFS rate of 61.4% and a 3‐year OS rate of 63.4% were achieved.^10^ In addition, Qi reported the survival outcomes of 1351 ENKTL patients based on nonanthracycline chemotherapy and presented a 5‐year OS rate of 68.9% and 5‐year PFS rate of 59.5%.^5^ In our study, we reported that chemotherapy with L‐asparaginase‐based regimens yielded an ORR of 83.5%, with a CR rate of 62.2%. The rates of 3‐year PFS and OS were 67.3% and 72.1%, respectively, and the rates of 5‐year PFS and OS were 64.3% and 68.5%, respectively. To our understanding, our study includes the largest dataset of ENKTL patients receiving specific L‐asparaginase‐based regimens, and the short‐term efficacy and long‐term survival results were analogous to those of the previously mentioned studies.

With the advent of L‐asparaginase, the survival outcomes of patients with ENKTL have improved. In a retrospective study developed among patients with stage II ENKTL, 48 patients receiving LOP (L‐asparaginase, vincristine, and dexamethasone) had statistically better short‐term efficacy (CR and ORR) and long‐term efficacy (OS and PFS) than patients who received the CHOP regimen.^14^ Ji et al reported that patients with newly diagnosed stage IV, relapsed, or refractory ENKTL who received the GLIDE regimen had a CR of 73.8% and an ORR of 83.3%.^15^ Jiang et al performed a prospective clinical study and reported that 66 newly diagnosed ENKTL patients treated with the LVDP regimen had an ORR of 92.4% with 83.3% CR.^7^ At the time of writing, no clinical data to compare the side effects and efficacies of the LVDP and GLIDE regimens have been found. In this retrospective analysis, we reported that the GLIDE group seemed to have a higher CR rate than the LVDP group. However, there were no significant differences between the LVDP group and GLIDE group, even after adjusting for confounders via PSM (CR, 57.6% vs. 65.8%, *p* = 0.264; ORR, 83.0% vs. 80.9%, *p* = 0.696; 5‐year PFS, 63.5% vs. 65.4%, *p* = 0.794; 5‐year OS, 67.7% vs. 67.6%, *p* = 0.867). In terms of toxicity, the GLIDE group had a higher incidence of myelosuppression and transaminase and fibrinogen abnormalities than the LVDP group. Treatment‐related infections causing death were reported; therefore, treatment‐associated myelosuppression should be handled carefully. In summary, considering its efficacy and toxicity, LVDP is a promising treatment option for ENKTL.

In addition, chemotherapy combined with radiotherapy plays an important role in ENKTL treatment.^16^ In the present study, 204 newly diagnosed ENKTL patients received chemoradiotherapy; of them, 94% of patients were early‐stage, and their survival outcomes were superior to those patients who were treated strictly through chemotherapy. This result was corroborate those of previous studies.^17^, ^18^ Vargo et al found that, during the initial treatment of early‐stage ENKTL, the omission of radiotherapy was associated with inferior survival.^18^ In a retrospective study, the early‐stage ENKTL patient cohort from the International T‐cell Lymphoma Project received chemoradiotherapy and achieved better survival outcomes than the cohort who received only chemotherapy (*p* = 0.045).^18^ In another multicenter study, Kwong et al. reported that 173 newly diagnosed early‐stage ENKTL patients received concomitant chemoradiotherapy, and the 5‐year OS rates stabilized around 72%–74%.^19^ Recently, a study reported that 202 early‐stage ENKTL patients received P‐Gemox combined with radiotherapy, and achieved a 3‐year PFS of 74.6% and a 3‐year OS of 85.2%.^20^ In this study, chemoradiotherapy yielded 73.6% of 5‐year OS rates for patients with ENKTL. Thus, our results further demonstrate the important role of radiotherapy in the treatment of ENTKL.

In many studies, AASS has been found to be an important prognostic factor for predicting PFS and OS in ENKTL. In 2006, Lee et al reported that the advanced stage (stage III/IV) was a vital prognostic factor for long‐term survival.^21^ Kim et al constructed a new prognostic model of ENKTL including advanced stage, and explored its role in guiding the treatment of patients with ENKTL.^22^ Recently, this prognostic model was further validated in asparaginase‐based chemotherapy.^20^ In our study, the advanced stage was an independent prognostic factor for OS by multivariate analysis, which was similar to prior studies.

Moreover, pretreatment hemoglobin has been considered to be a vital prognostic factor for predicting the PFS and OS of ENKTL. In a previous study, 32% of patients with NHL had anemia, and the incidence of anemia varied by lymphoma subtypes, with a lower frequency of indolent lymphomas and a higher frequency of aggressive lymphomas such as T‐cell lymphomas.^23^ Some researches have demonstrated that radiotherapy and chemotherapy might be more effective in a well‐oxygenated tumor microenvironment than hypoxia.^24^ Wang et al reported that the anemia status before treatment could affect the efficacy of radiotherapy and chemotherapy and consequently lead to inferior PFS and OS.^25^ In the present study, we reported that Hb <110 g/L was an independent prognostic factor for PFS, which was in line with previous studies. As cases with anemia are especially uncommon, additional exploration is required to validate its value in prognosis for ENKTL.

However, a number of limitations affected this study. First, as this was a single‐center retrospective study, a degree of selection bias was likely present. Second, patients with ECOG‐PS score ≥2 and advanced‐stage patients were comparatively fewer, and this might impact the prognostic analysis. Third, the individual chemoradiotherapy schedules for ENKTL patients in our data varied, and this might cause additional bias in survival outcomes. Considering these limitations, subsequent multicenter randomized controlled trials should be conducted to further support and confirm the results presented in this study.

## 5 CONCLUSION

In conclusion, LVDP and GLIDE regimens are effective for ENKTL. However, treatment‐related toxicities were more frequently observed in the GLIDE group than in the LVDP group. Our study provides substantial evidence that LVDP has the potential as an efficacious treatment regimen for ENKTL.

## AUTHOR CONTRIBUTIONS

**Wanchun Wu:** Conceptualization (equal); data curation (lead); formal analysis (lead); writing – original draft (lead). **Kexin Ren:** Data curation (equal); formal analysis (equal); writing – review and editing (equal). **Xi Chen:** Formal analysis (equal); writing – review and editing (equal). **Na Li:** Data curation (equal); writing – original draft (equal). **Qian Luo:** Writing – original draft (supporting). **Tao Hai:** Writing – review and editing (supporting). **Huijie Zhou:** Data curation (supporting). **Liqun Zou:** Conceptualization (lead); supervision (lead).

## FUNDING INFORMATION

No funding was received for this study.

## CONFLICT OF INTEREST STATEMENT

The authors have no relevant financial or non‐financial interests to disclose.

## ETHICS APPROVAL STATEMENT

Ethical approval was obtained from the Ethics Committee of West China Hospital (ID of ethics approval: SCHX‐2022‐64).

## Supporting information


Table S1

## Data Availability

The data that support the findings of this study are available from the corresponding author upon reasonable request.
