# Supplementary material for: Comparison of the clinical efficacies of two L‐asparaginase‐based chemotherapy regimens for newly diagnosed nasal‐type extranodal NK/T‐cell lymphoma
Source: Cancer Med. 2023 Mar 31;12(8):9458–70. doi: 10.1002/cam4.5708 (PMC10166983; doi:10.1002/cam4.5708)
Supplement: Supplementary file 1 — Table S1 [file CAM4-12-9458-s001.docx]

Table S1. The short-term efficacy of the two regimens for early-stage and advanced-stage patients before and after propensity score matching

|  | | **Total**  **(n=267)**  **(%)** | | **Before PSM** | | | | | | |  | **After PSM** | | | | | | | |
| --- | --- | --- | --- | --- | --- | --- | --- | --- | --- | --- | --- | --- | --- | --- | --- | --- | --- | --- | --- |
|  |  |  |  | **LVDP**  **(n=160)**  **(%)** | **GLIDE**  **(n=53)**  **(%)** |  | **LVDP**  **(n=25)**  **(%)** | **GLIDE**  **(n=29)**  **(%)** |  |  | | **LVDP**  **(n=95)**  **(%)** | | **GLIDE**  **(n=53)**  **(%)** |  | **LVDP**  **(n=23)**  **(%)** | **GLIDE**  **(n=20)**  **(%)** | |  |
|  | Stage  Ⅰ/Ⅱ | | Stage  Ⅲ/Ⅳ | Stage  Ⅰ/Ⅱ | Stage  Ⅰ/Ⅱ | **p** | Stage  Ⅲ/Ⅳ | Stage  Ⅲ/Ⅳ | **p** |  | | Stage  Ⅰ/Ⅱ | Stage  Ⅰ/Ⅱ | | **p** | Stage  Ⅲ/Ⅳ | Stage  Ⅲ/Ⅳ | **p** | |
| CR | 149(70) | | 17(31.5) | 111(69.4) | 38(71.7) | 0.749 | 4(16) | 13(44.8) | 0.023 |  | | 64(67.4) | 38(71.7) | | 0.585 | 4(17.4) | 10(50) | 0.023 | |
| PR | 42(19.7) | | 15(27.8) | 34(21.3) | 8(15.1) | - | 9(26) | 6(20.7) | - | |  | 22(23.2) | 8(15.1) | | - | 8(34.8) | 3(15) | - | |
| SD | 4(1.9) | | 4(7.4) | 1(0.6) | 3(5.7) | - | 2(8) | 2(6.9) | - |  | | 1(1.1) | 3(5.7) | | - | 2(8.7) | 2(1) | - | |
| PD | 18(8.4) | | 18(33.3) | 14(25.9) | 4(7.5) | - | 10(40) | 8(27.6) | - |  | | 8(8.4) | 4(7.5) | | - | 9(39.1) | 5(25) | - | |
| ORR | 191(89.8) | | 32(59.3) | 145(90.6) | 46(86.8) | 0.427 | 13(52) | 19(65.5) | 0.313 |  | | 86(90.5) | 46(86.8) | | 0.483 | 12(52.2) | 13(65) | 0.395 | |

Abbreviation: CR, complete response; GLIDE, gemcitabine, L-asparaginase, ifosfamide, dexamethasone and etoposide; LVDP, L-asparaginase, etoposide, dexamethasone and cisplatin; ORR, objective response rate; PD, progressive disease; PR, partial response; SD, stable disease.
